# Supplementary material for: Analysis of PPARγ Signaling Activity in Psoriasis
Source: Int J Mol Sci. 2021 Aug 10;22(16):8603. doi: 10.3390/ijms22168603 (PMC8395241; doi:10.3390/ijms22168603)
Supplement: Supplementary file 1 [file ijms-22-08603-s001.zip › Supplemental materials_Analysis of PPARg signaling activity in psoriasis/Pathway models/Models images and html files/Interleukin-17 and interleukin-22 signaling in psoriasis/15908.html]

TRAF3IP2


# Protein TRAF3IP2

|  |  |
| --- | --- |
| URN | urn:agi-llid:10756 |
| Total Entities | 0 |
| Connectivity | 530 |
| Name | TRAF3IP2 |
| Description | TRAF3 interacting protein 2 |
| Notes | This gene encodes a protein involved in regulating responses to cytokines by members of the Rel/NF-kappaB transcription factor family. These factors play a central role in innate immunity in response to pathogens, inflammatory signals and stress. This gene product interacts with TRAF proteins (tumor necrosis factor receptor-associated factors) and either I-kappaB kinase or MAP kinase to activate either NF-kappaB or Jun kinase. Several alternative transcripts encoding different isoforms have been identified. Another transcript, which does not encode a protein and is transcribed in the opposite orientation, has been identified. Overexpression of this transcript has been shown to reduce expression of at least one of the protein encoding transcripts, suggesting it has a regulatory role in the expression of this gene. [provided by RefSeq, Aug 2009] |

---

|  |  |
| --- | --- |
| Pathway | Proteins Involved in Myocardial Ischemia |
|  | IL17 Signaling in Psoriasis |
|  | Genes with Mutations Associated with Psoriasis |
|  | Il17 Signaling Related Neutrophilia in Asthma |
|  | IL17F Signaling in Bronchial Epithelial Cell in Asthma |
|  | Synovial Fibroblast Activation in Psoriatic Arthritis |
|  | Keratinocyte Activation in Psoriatic Arthritis |
|  | Cytokines Trigger Otitis Media |
|  | IL23A and IL17A Provoke Cancer-Associated Inflammation |
|  | 2\_Interleukin-17 and interleukin-22 signaling in psoriasis |
|  | 3\_2\_Regulation of airway surface liquid (ASL) thickness |
|  | 4\_1\_Polymorphisms associated with inflammatory bowel diseases |
|  | Interleukin-17 and interleukin-22 signaling in psoriasis |

---

|  |  |
| --- | --- |
| Group | Genes with Mutations Associated with Psoriasis |
|  | Genes with Mutations Associated with Psoriatic Arthritis |

---

|  |  |
| --- | --- |
| MedScan ID | 10756 |

---

|  |  |
| --- | --- |
| LocusLink ID | 10756 |
|  | 10758 |
|  | 103213 |
|  | 361857 |

---

|  |  |
| --- | --- |
| Alias | OTTHUMP00000017022 |
|  | RP3-487J7.3 |
|  | Ciks |
|  | C6orf2 |
|  | traf3ip2 |
|  | OTTHUMP00000017024 |
|  | NFkB-activating protein ACT I |
|  | chromosome 6 open reading frame 6 |
|  | C6orf5 |
|  | MGC3581 |
|  | AI429613 |
|  | chromosome 6 open reading frame II |
|  | CANDF8 |
|  | chromosome 6 open reading frame 5 |
|  | ACT1 |
|  | traf3 interacting protein 2 |
|  | NFkB-activating protein ACT1 |
|  | traf3 interacting protein II |
|  | chromosome 6 open reading frame V |
|  | DKFZP586G0522 |
|  | PSORS13 |
|  | nuclear factor NF-kappa-B activator 1 |
|  | C6orf6 |
|  | RGD1304768 |
|  | C6orf4 |
|  | chromosome 6 open reading frame 4 |
|  | chromosome 6 open reading frame IV |
|  | chromosome 6 open reading frame 2 |
|  | MGC112844 |
|  | connection to IKK and SAPK/JNK |
|  | ACT I |
|  | OTTHUMP00000040422 |
|  | adaptor protein CIKS |
|  | adapter protein CIKS |

---

|  |  |
| --- | --- |
| GO ID | 0005102 |
|  | 0001783 |
|  | 0006959 |
|  | 0048305 |
|  | 0035556 |
|  | 0043123 |
|  | 0002230 |
|  | 0005622 |
|  | 0005623 |

---

|  |  |
| --- | --- |
| KEGG ID | hsa:10758 |

---

|  |  |
| --- | --- |
| Organism | Homo sapiens {Organism urn:agi-taxid:9606} |
|  | Mus musculus {Organism urn:agi-taxid:10090} |
|  | Rattus norvegicus {Organism urn:agi-taxid:10116} |
|  | Homo sapiens |
|  | Mus musculus |
|  | Rattus norvegicus |

---

|  |  |
| --- | --- |
| Mouse chromosome position | 10 |

---

|  |  |
| --- | --- |
| OMIM ID | 607043 |
|  | 615527 |
|  | 614070 |

---

|  |  |
| --- | --- |
| Rat chromosome position | 20q12 |

---

|  |  |
| --- | --- |
| Hugo ID | 1343 |
|  | HGNC:1343 |

---

|  |  |
| --- | --- |
| Human chromosome position | 6q21 |

---

|  |  |
| --- | --- |
| Swiss-Prot Accession | O43734 |
|  | O43734.3 |
|  | Q8N7N6 |
|  | Q498R8 |
|  | B2RAY9 |
|  | E1P555 |
|  | Q5R3A3 |
|  | Q7Z6Q1 |
|  | Q7Z6Q2 |
|  | Q7Z6Q3 |
|  | Q9H5W2 |
|  | Q9H6Y3 |
|  | Q9NS14 |
|  | Q9UG72 |

---

|  |  |
| --- | --- |
| PIR ID | T08794 |

---

|  |  |
| --- | --- |
| GenBank ID | NC\_000006 |
|  | NM\_001164281 |
|  | NP\_001157753 |
|  | NM\_147686 |
|  | NP\_679211 |
|  | NM\_147200 |
|  | NP\_671733 |
|  | NM\_001164283 |
|  | NP\_001157755 |
|  | NG\_032030 |
|  | AL008730 |
|  | CH471051 |
|  | EAW48285 |
|  | EAW48286 |
|  | EAW48287 |
|  | EAW48288 |
|  | CS135278 |
|  | CAJ21527 |
|  | Z97989 |
|  | AF085921 |
|  | AF136405 |
|  | AAF67445 |
|  | AF136406 |
|  | AAF67446 |
|  | AF136407 |
|  | AAF67447 |
|  | AF272151 |
|  | AAG15407 |
|  | AF274303 |
|  | AAG15367 |
|  | AK025351 |
|  | BAB15117 |
|  | AK026602 |
|  | BAB15507 |
|  | AK310141 |
|  | AK310922 |
|  | AK314415 |
|  | BAG37036 |
|  | AL050289 |
|  | CAB43390 |
|  | AW296296 |
|  | AW513823 |
|  | BC002823 |
|  | AAH02823 |
|  | BC026723 |
|  | BE904103 |
|  | BI856094 |
|  | BM449775 |
|  | CB049654 |
|  | DA130363 |
|  | DA665549 |
|  | DA836923 |
|  | DC348710 |
|  | DT217829 |
|  | O43734 |
|  | NC\_000076 |
|  | NM\_134000 |
|  | NP\_598761 |
|  | XM\_011243105 |
|  | XP\_011241407 |
|  | AC160403 |
|  | CH466540 |
|  | EDL04930 |
|  | KC414133 |
|  | AB238206 |
|  | BAF34919 |
|  | AB238207 |
|  | BAF34920 |
|  | AK031556 |
|  | BAC27449 |
|  | AK037407 |
|  | BAC29801 |
|  | AK052190 |
|  | BAC34875 |
|  | AK098107 |
|  | BAC05236 |
|  | BC008534 |
|  | BC008535 |
|  | BC022633 |
|  | AAH22633 |
|  | BC071207 |
|  | AAH71207 |
|  | BC096483 |
|  | AAH96483 |
|  | BC138234 |
|  | AAI38235 |
|  | BC138235 |
|  | AAI38236 |
|  | JX869941 |
|  | AFY98903 |
|  | NC\_005119 |
|  | XR\_597418 |
|  | XR\_597419 |
|  | NM\_001044248 |
|  | NP\_001037713 |
|  | XM\_006256532 |
|  | XP\_006256594 |
|  | AC\_000088 |
|  | AABR07045351 |
|  | AABR07045352 |
|  | AAHX01101535 |
|  | AAHX01101536 |
|  | AAHX01101537 |
|  | CH474051 |
|  | EDL87824 |
|  | EDL87825 |
|  | BC100099 |
|  | AAI00100 |
|  | NR\_028338 |
|  | XM\_011535386 |
|  | XP\_011533688 |
|  | XM\_006715319 |
|  | XP\_006715382 |
|  | NC\_018917 |
|  | AMYH02014306 |
|  | JF432241 |
|  | ADZ15458 |
|  | AC\_000032 |
|  | AAHY01084885 |
|  | AAHY01084886 |
|  | AAHY01084887 |
|  | AAHY01084888 |
|  | XM\_006256533 |
|  | XP\_006256595 |
|  | AC\_000138 |
|  | ABBA01026085 |
|  | ABBA01026086 |
|  | XM\_006512495 |
|  | XP\_006512558 |

---

|  |  |
| --- | --- |
| Swiss-Prot ID | CIKS\_HUMAN |

---

|  |  |
| --- | --- |
| Ensembl ID | ENSG00000056972 |
|  | ENSP00000352889.4 |
|  | ENST00000359831.8 |
|  | ENSP00000357724.1 |
|  | ENST00000368735.1 |
|  | ENSP00000345984.6 |
|  | ENST00000340026.10 |
|  | ENSP00000357750.5 |
|  | ENST00000368761.10 |
|  | ENSMUSG00000019842 |
|  | ENSMUSP00000019987.6 |
|  | ENSMUST00000019987.6 |
|  | ENSRNOG00000000595 |
|  | ENSRNOP00000000728.5 |
|  | ENSRNOT00000000728.5 |
|  | ENSP00000352889 |
|  | ENST00000359831 |
|  | ENSP00000357724 |
|  | ENST00000368735 |
|  | ENSP00000357750 |
|  | ENST00000368761 |
|  | ENSP00000345984 |
|  | ENST00000340026 |
|  | ENSMUSP00000019987 |
|  | ENSMUST00000019987 |
|  | ENSRNOP00000000728 |
|  | ENSRNOT00000000728 |

---

|  |  |
| --- | --- |
| MGI ID | MGI:2143599 |

---

|  |  |
| --- | --- |
| RGD ID | 1593240 |

---

|  |  |
| --- | --- |
| Unigene ID | Hs.561514 |
|  | Hs.740602 |
|  | Mm.436686 |
|  | Rn.9031 |

---

|  |  |
| --- | --- |
| Homologene ID | 15885 |

---
